# Association of serum glycine levels with metabolic syndrome in an elderly Chinese population

**DOI:** 10.1186/s12986-018-0325-4

**Published:** 2018-12-17

**Authors:** Xianghui Li, Liang Sun, Wenduo Zhang, Hongxia Li, Siming Wang, Hongna Mu, Qi Zhou, Ying Zhang, Yueming Tang, Yu Wang, Wenxiang Chen, Ruiyue Yang, Jun Dong

**Affiliations:** 1 Peking University Fifth School of Clinical Medicine, The MOH Key Laboratory of Geriatrics, Beijing Hospital, National Center of Gerontology, Beijing, 100730 People’s Republic of China; 2 Department of Cardiology, Beijing Hospital, Beijing, 100730 People’s Republic of China

**Keywords:** Serum glycine, Metabolic syndrome, Elderly

## Abstract

**Background:**

Several studies have identified a negative association between serum glycine (Gly) levels and metabolic syndrome (MetS). However, this association has not been fully established in the elderly.

**Methods:**

A total of 472 Chinese individuals (272 males and 200 females, 70.1 ± 6.6 years old) participated in a population-based, cross-sectional survey in Beijing Hospital. The MetS and its components were defined based on the 2006 International Diabetes Federation (IDF) standard for Asians. Serum Gly concentration was determined using isotope dilution liquid chromatography tandem mass spectrometry.

**Result:**

The proportion of patients with MetS decreased gradually with increasing Gly levels (*p* for trend < 0.001), and serum Gly concentrations declined gradually with increasing numbers of MetS components (*p* = 0.03 for trend). After adjusting for age and gender, lower Gly levels were significantly associated with MetS and central obesity, with OR (95% CI) of 0.40 (0.25–0.65) and 0.46 (0.28–0.74). The stratified analysis conducted according to age showed that the OR between serum Gly levels and MetS was greater in those older than 65 (OR = 0.66; 95% CI, 0.51–0.86) than in those younger than 65 (OR = 0.89; 95% CI, 0.54–1.46). In the stratified analysis, using other age cut-off points, the results consistently showed that the association between serum Gly levels and MetS was more remarkable in the older groups.

**Conclusions:**

Gly levels are associated with cardiometabolic characteristics and MetS in the elderly, and the association is more pronounced in very old people than in younger old people.

## Introduction

In recent years, the improvements in living standards, economic development, and lifestyle changes have led to a longer life expectancy for older people, and the global elderly population has continued to increase. With the increase of age, the incidence of cardiometabolic diseases in the elderly may rise gradually [1, 2]. However, most studies of the metabolic and cardiovascular risk factors have focused on young to middle-aged adults, and the impact of most risk factors on diseases may generally change with aging [3]. Due to the special physiological characteristics of the elderly, it is not appropriate to apply these risk factor assessment criteria to the elderly population.

Metabolic syndrome (MetS) is reported to be a risk factor for increased risk of type 2 diabetes and cardiovascular disease and a proven risk factor for cardiovascular morbidity, especially stroke and coronary heart disease (CHD), and mortality in the elderly [4, 5]. According to the International Diabetes Federation (IDF), MetS is highly prevalent in old people, in whom it varies from 37 to 41.9% [6]. Given that digestive and metabolic functions are gradually weakened in the elderly, eventually leading to reduced intake and absorption of nutrients, catabolism plays a dominant role in the metabolic processes of the elderly. Compared to young adults, amino acids and other nutrients may play a more important role in the elderly, and their levels in serum may mostly reflect nutritional status rather than metabolic disorder [7]. With the development of metabolomics technology, it has become an important way to evaluate nutritional level and disease risk. Recent metabolomics studies have found the relationship between serum levels of amino acids such as glycine (Gly) and cardiometabolic disease.

Gly is a major and the simplest non-essential amino acid in humans, animals, and many mammals. It is mainly generated in the liver and kidney and is used to produce creatine, purine, glucose, and collagen and is involved in anti-inflammatory processes, the immune function, and anti-oxidation reactions [8]. Recently, some studies have demonstrated the inverse association of serum Gly levels with several traditional cardiovascular risk factors such as hypertension [9], type 2 diabetes (T2D) [10], and obesity [11]. Researchers have found that circulation Gly levels in patients with MetS are significantly lower than in normal subjects [12, 13]. Moreover, serum Gly levels in obese and diabetic patients are significantly lower than those in healthy individuals, and the improvement of insulin resistance can increase serum Gly levels [14–16]. However, the associations of serum Gly levels with MetS and its components have not been fully established in the elderly.

Therefore, we proposed to use a cross-sectional study of 472 Chinese subjects, of whom about 80% are older than 65 years old, to investigate the association between serum Gly levels and MetS in the elderly and whether this association in the elderly is different from relatively young adults.

## Methods

### Study population

The design of and recruitment for the population-based cross-sectional study has been described in detail elsewhere [17]. Briefly, a total of 472 eligible participants (272 males and 200 females, 70.1 ± 6.6 years old) from a group of Beijing residents attending an annual physical examination in a period from August to October, 2011, were recruited randomly. People with malignant tumors, blood system diseases, chronic obstructive pulmonary disease, autoimmune diseases, infectious diseases, and have taking amino acid supplements were excluded. The sera from fasting blood samples obtained from subjects were isolated and stored at − 80 °C until being analyzed. This study was approved by the Ethics Committee of the Beijing Hospital of the Ministry of Health, and written, informed consents were obtained from all participants.

### Serum Gly measurement

Serum Gly concentrations were determined using isotope dilution liquid–chromatography tandem mass spectrometry. Briefly, 0.05 ml aliquots of Gly (Sigma-Aldrich, USA) calibrators (539.8, 269.9, 134.9, 67.5, and 33.7 μmol/l) or serum samples were mixed with 0.05 ml of the isotope-labeled internal standard (Gly-D_5_, Cambridge Isotope Laboratories, USA) solution (312.5 μmol/l). Serum Gly was extracted with 0.4 ml of acetonitrile containing 0.1% formic acid and analyzed using liquid chromatography method associated with mass spectrometry (LC-MS/MS). The LC separation was performed on an Agilent 1200 series LC system. Two microliter aliquots of the prepared samples were injected onto a Waters Shield C18 column (3.5 μm, 2.1 × 150 mm) maintained at 20 °C and eluted with a mobile phase of 0.01% formic acid in water-acetonitrile (90:10) at a flow rate of 0.3 ml/min. An API 4000 triple quadrupole mass spectrometer (Sciex Applied Biosystems) was used for the MS/MS detection. The detection was performed with positive electronic spray ionization (ESI) in multiple reaction monitoring (MRM) mode at a source temperature of 700 °C and a voltage of 5500 V. The dwell times were 0.08 s for MRM. Nitrogen was used as the curtain, nebulizer, and collision gas at pressures of 50, 60, and 70 psi, respectively. Certain ion transitions for Gly and the internal standards were m/z 76.0 → 30.0 and 78.0 → 32.0, respectively. The calibration curve was generated using a linear regression of the peak area ratios (y) versus the amino acid concentrations (x) of the calibrators.

### Other parameters and laboratory assays

Information about demographic variables—health status, smoking and alcohol drinking status, medications, and family history of diseases—were recorded using a standardized questionnaire. Height, body weight, waist circumference (WC), and sitting blood pressure (BP) were measured. The serum samples were also tested for fasting blood glucose (FBG), total cholesterol (TC), triglyceride (TG), high-density lipoprotein cholesterol (HDL-C), low-density lipoprotein cholesterol (LDL-C), apolipoprotein (apo) AI and B, and high-sensitive C-reactive protein (CRP), using assay kits from Sekisui Medical Technologies (Osaka, Japan) on a Hitachi 7180 chemistry analyzer.

### Definition of MetS

MetS and its components were defined based on the 2006 International Diabetes Federation standard for Asians with central obesity (male: WC ≥90 cm; female: WC ≥80 cm), in addition to two or more of the following criteria [18]: (1) BP ≥130/85 mmHg or taking antihypertensive drugs; (2) TG ≥1.7 mmol/L (150 mg/dl) or had received treatment; (3) HDL-C: male < 1.04 mmol/L (40 mg/dl), female < 1.3 mmol/L (50 mg/dl) or had received treatment; (4) FPG ≥5.6 mmol/L (100 mg/dl) or had been diagnosed with type 2 diabetes.

### Statistical analysis

The main continuous variables were all tested for normality. Normally distributed data were expressed as means ± SD, whereas variables obeying a skewed distribution were shown as median (interquartile range). Count data were reported as frequencies and percentages. For quantitative data, the variance analysis was conducted to compute the difference across the Gly tertiles, whereas for count data, the Chi-square test was used. Correlation coefficients between Gly levels and metabolic traits were calculated by Spearman partial correlation after adjustment for age and gender. Serum Gly levels were depicted according to the number of MetS components, using a linear regression model. Multivariate logistic regression models were used to estimate the odds ratios (ORs) and 95% confidence intervals (CIs), which adopted the entry method and the maximum likelihood ratio test for MetS and its components. Potential confounding variables, including age; gender; smoking; alcohol drinking; family history of diabetes, hypertension, and CAD; and CRP, were controlled in the regression models. The performance of Gly measurements for classifying MetS status was assessed by logistic regression and receiver operating characteristic analysis. Stratified analyses were performed according to age and serum CRP levels [< 0.09 (median) and ≥ 0.09 mg/dL]. Two-sided *P* values < 0.05 were considered statistically significant. SPSS 21.0 software (SPSS Inc.) was used for statistical analysis of the data, and PASS 15.0 software was used for calculating the statistical power.

## Result

### Characteristics of population according to Gly tertile

The Gly levels of 472 subjects had skewed distribution (Skewness = 1.95, kurtosis = 6.53), and they could not be normally distributed after natural logarithm, logarithmic transformation of 10, and square root (SQRT) transformation. The prevalence of metabolic syndrome in our study was 39.0%. As shown in Table 1, according to serum Gly tertile levels, the subjects with higher Gly levels were more likely to be female (*p* < 0.05). With respect to metabolic traits, BMI, WC, and TG significantly declined with increasing Gly levels, which had a dose–response relationship (all *p* < 0.01 for trend); in contrast, HDL-C levels increased notably (*p* < 0.01 for trend). However, increased Gly levels did not show association with changes in BP, TC, LDL-C, andCRP levels. Remarkably, the proportion of patients with MetS decreased gradually with increasing Gly levels (*p* for trend < 0.001).

**Table 1:** Characteristics of the study population by serum Gly tertiles

Characteristic^a^	Tertile of serum Gly			*P*_trend_^b^
	T1	T2	T3	
Number	158	157	157	
Gly, μmol/L	219.3 (205.2–230.8)	253.5 (247.2–261.2)	301.1 (285.0–344.7)	< 0.001
Age, years	70.6 ± 6.7	69.9 ± 6.1	69.7 ± 6.9	0.253
Male, n (%)	102 (64.6)	91 (57.9)	79 (50.3)	0.011
BMI, kg/m^2^	25.0 ± 3.2	24.4 ± 3.2	23.7 ± 3.4	0.001
WC, cm	88.6 ± 8.4	87.4 ± 9.2	84.4 ± 8.8	< 0.001
SBP, mmHg	134.8 ± 16.3	136.6 ± 19.9	132.8 ± 16.9	0.331
DBP, mmHg	76.8 ± 8.0	75.7 ± 9.1	75.8 ± 8.0	0.341
Current smoker, n (%)	11 (7.0)	17 (10.8)	15 (9.6)	0.424
Current drinker, n (%)	20 (12.7)	25 (15.9)	15 (9.6)	0.901
FBG, mmol/L	5.8 ± 1.1	5.8 ± 1.3	5.6 ± 0.9	0.164
TG, mmol/L	1.4 (1.0–2.0)	1.3 (0.9–1.7)	1.2 (0.8–1.6)	0.001
TC, mmol/L	4.9 ± 1.0	5.0 ± 1.0	5.0 ± 0.8	0.312
HDL-C, mmol/L	1.3 (1.1–1.5)	1.3 (1.1–1.6)	1.4 (1.2–1.6)	0.004
LDL-C, mmol/L	2.9 (2.3–3.5)	3.0 (2.3–3.5)	2.9 (2.4–3.3)	0.708
Apo AI, mg/dL	138.6 (127.3–149.2)	137.5 (128.7–150.6)	142.0 (130.6–153.5)	0.075
ApoB, mg/dL	98.8 ± 21.2	98.3 ± 22.3	97.2 ± 19.7	0.499
CRP, mg/dL	0.1 (0.05–0.18)	0.1 (0.05–0.19)	0.08 (0.04–0.15)	0.067
MetS^c^, n (%)	78 (49.4)	59 (37.6)	47 (29.9)	< 0.001

*Abbreviations*: *Gly* glycine, *BMI* body mass index, *WC* waist circumference, *SBP* systolic blood pressure, *DBP* diastolic blood pressure, *FBG* fasting blood glucose, *TC* total cholesterol, *TG* triglyceride, *HDL-C* high-density lipoprotein cholesterol, *LDL-C* low-density lipoprotein cholesterol, *MetS* metabolic syndrome, *CRP* high-sensitivity C-reactive protein. Apo AI, B, CII, and CIII represent apolipoprotein AI, B, CII, and CIII, respectively

^a^Data are mean ± SD, median (interquartile range) for continuous variables, or percentage for categorical variables

^b^*P* values for trend

^c^Defined according to the criteria for metabolic syndrome from the International Diabetes Federation (IDF) (2006) for Asians

**Fig. 1:**
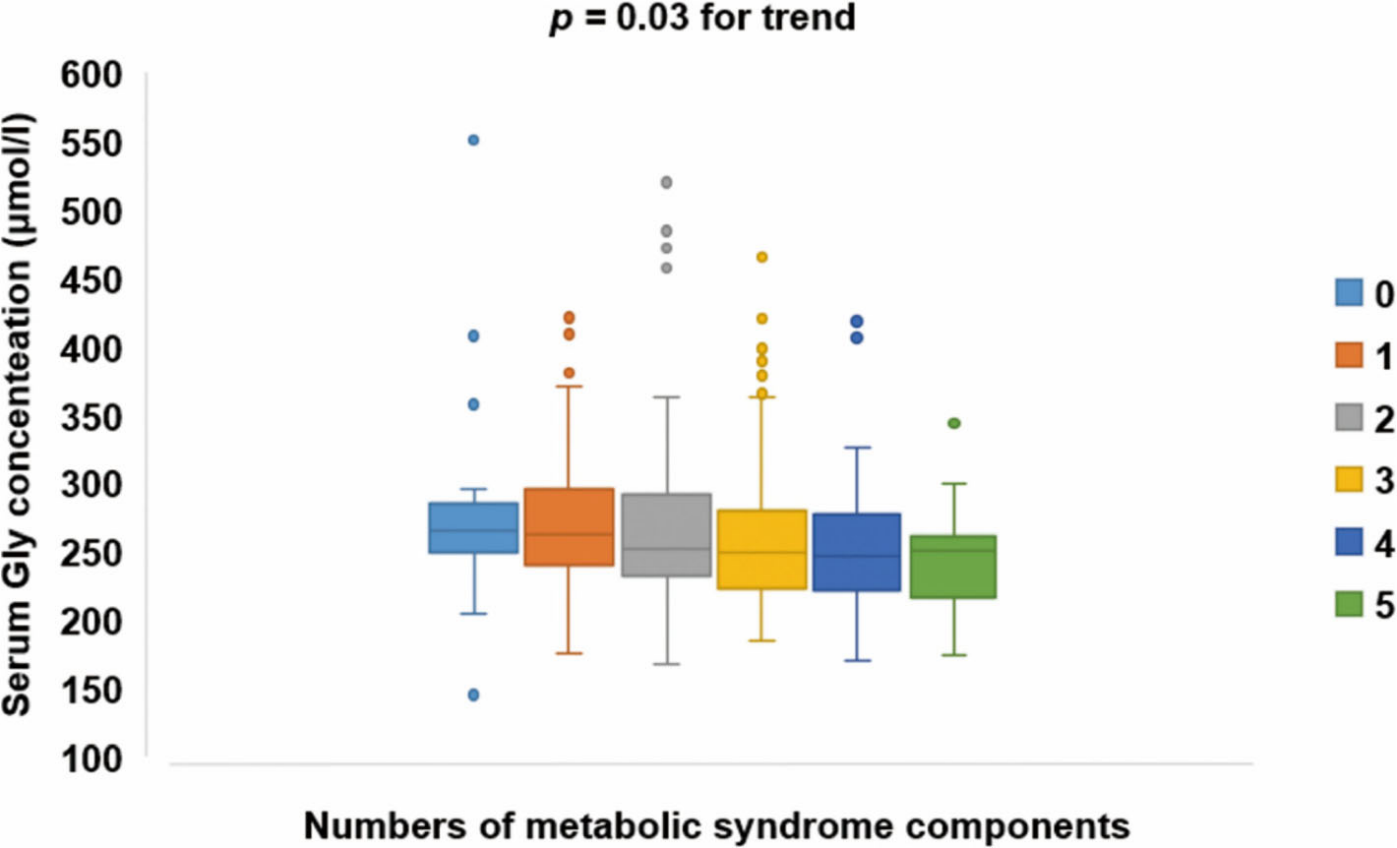
Box-chart for serum Gly concentrations according to the number of MetS components. Data are depicted by box plots extending from the 25th to the 75th percentile and whiskers ranging from the lower limit to the upper limit. The horizontal line in the box plot represents the median, and dots in the figure represent outliers. MetS and its components were defined based on the 2006 IDF standard. Serum Gly concentrations declined gradually with an increasing number of MetS components (*p* = 0.03 for trend)

Serum Gly concentrations declined gradually with increasing numbers of MetS components (*p* = 0.03 for trend) (Fig. 1). The median (interquartile range) of serum Gly concentrations for those with none to five components were 265.8 (247.1–290.9), 262.6 (240.4–296.1), 252.0 (232.1–293.1), 249.9 (223.1–280.9), 247.1 (220.5–281.2), 250.7 (214.5–265.4) μmol/L, respectively.

**Fig. 2:**
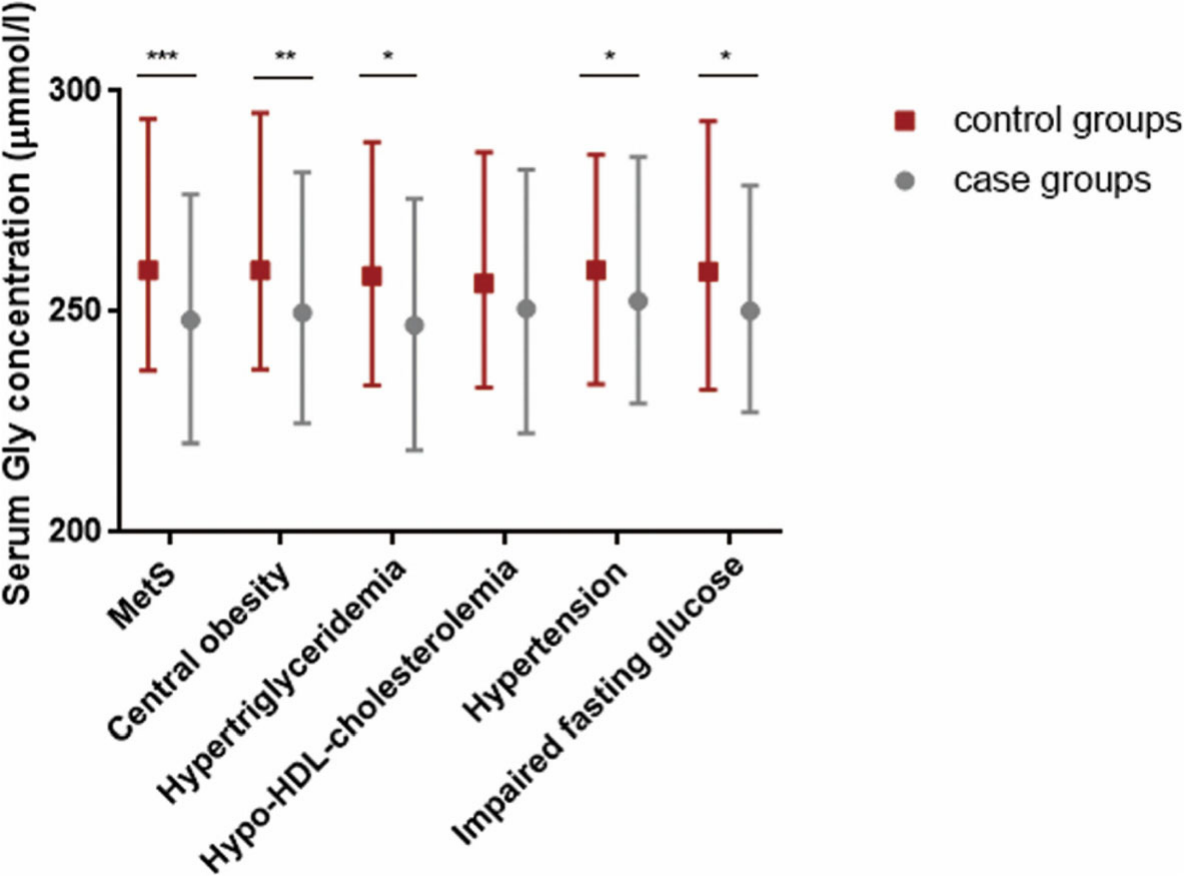
Comparison of the circulating Gly concentrations median (interquartile range) according to MetS and its components. MetS and its components were defined based on the 2006 IDF standard. The Mann-Whitney U rank-sum test was used to estimate the difference between two groups. *p < 0.05, **p < 0.01, ***p < 0.001

According to the definition of MetS, the population was divided into control and case groups, respectively, based on whether the population had MetS, abdominal obesity, hypertriglyceridemia, hypo-HDL-cholesterolemia, hypertension, and impaired fasting glucose (IFG). Regarding the patterns of dichotomous cardiometabolic traits, serum Gly levels were found to be lower in subjects with MetS, central obesity, hypertriglyceridemia, hypertension, and IFG than in the corresponding control groups (*p* < 0.05) (Fig. 2). No difference existed between serum Gly levels and hypo-HDL-cholesterolemia.

### Correlations between serum Gly levels and cardiometabolic risk factors

As shown in Table 2, partial Spearman correlation analysis demonstrated the correlation between metabolic characteristics and Gly concentration. The strongest correlation was between Gly and BMI after adjusting for age and gender (*p* < 0.001, *R* = − 0.197). Serum Gly concentration was significantly negatively associated with most of the cardiovascular risk factors, such as BMI, WC, TG, and CRP (*p* < 0.05) and were positively associated with protective factors, including HDL-C (*p* < 0.01).

**Table 2:** Partial correlation coefficients between serum Gly concentration and metabolic characteristics after adjustment for age and gender

	Total R	*P*
BMI	−0.197	< 0.001
WC	−0.193	< 0.001
SBP	−0.087	0.065
DBP	−0.054	0.249
FBG	−0.083	0.078
TG	−0.142	0.002
TC	0.004	0.927
HDL-C	0.141	0.003
LDL-C	−0.028	0.555
Apo AI	0.079	0.093
ApoB	−0.075	0.109
CRP	−0.093	0.049

All correlation coefficients were calculated after adjustment for age and gender

*Abbreviations*: *Gly* glycine, *BMI* body mass index, *WC* waist circumference, *SBP* systolic blood pressure, *DBP* diastolic blood pressure, *FBG* fasting blood glucose, *TC* total cholesterol, *TG* triglyceride, *HDL-C* high-density lipoprotein cholesterol, *LDL-C* low-density lipoprotein cholesterol, *CRP* high-sensitivity C-reactive protein. Apo AI, B represent apolipoprotein AI and B, respectively

### Association of serum Gly levels with MetS and its components

As presented in Table 3, the associations between serum Gly levels and MetS and its components were analyzed by multivariate logistic regression. The ORs for MetS, central obesity, and hypertriglyceridemia were all meaningful no matter whether serum Gly levels were regarded as continuous variables or categorical variables (all *p* < 0.05). When Gly levels were used as categorical variables, the crude ORs for MetS, central obesity, and hypertriglyceridemia were lower with increasing Gly levels in model 1, which had a dose–response relationship (*p* < 0.05 for trend). After adjusting for age and gender (model 2), lower Gly levels were significantly associated with MetS and central obesity, with ORs (95% CI) of 0.40 (0.25–0.65) and 0.46 (0.28–0.74). Further controlling for smoking status; alcohol drinking; CRP; and family history of diabetes, hypertension, and CAD only slightly attenuated the strength of the association for MetS, central obesity, and hypertriglyceridemia. The results were the same when the Gly levels were used as a continuous variable. The sensitivity and specificity of Gly measurements for MetS diagnosis were 71.2 and 65.5%, respectively.

**Table 3:** Odds ratios (95% confidence intervals) for MetS and its components according to serum Gly levels

	Gly as a continuous variable		Gly as a categorical variable			
	Per SD increment	*P*	T1	T2	T3	*P*_trend_^a^
MetS						
Model 1 ^b^	0.75 (0.61–0.93)	0.008	1.0	0.61 (0.39–0.97)	0.43 (0.27–0.70)	< 0.001
Model 2 ^c^	0.71 (0.58–0.88)	0.002	1.0	0.59 (0.37–0.94)	0.40 (0.25–0.65)	< 0.001
Model 3 ^d^	0.72 (0.57–0.91)	0.005	1.0	0.66 (0.40–1.09)	0.43 (0.26–0.71)	0.001
Central obesity						
Model 1 ^b^	0.79 (0.65–0.95)	0.013	1.0	0.58 (0.37–0.93)	0.54 (0.34–0.87)	0.011
Model 2 ^c^	0.69 (0.56–0.84)	0.0001	1.0	0.54 (0.33–0.87)	0.46 (0.28–0.74)	0.002
Model 3 ^d^	0.68 (0.54–0.84)	0.001	1.0	0.62 (0.37–1.03)	0.49 (0.29–0.81)	0.006
Hypertriglyceridemia						
Model 1 ^b^	0.83 (0.67–1.04)	0.109	1.0	0.64 (0.39–1.04)	0.48 (0.29–0.80)	0.004
Model 2 ^c^	0.77 (0.61–0.97)	0.024	1.0	0.60 (0.37–0.99)	0.42 (0.25–0.71)	< 0.001
Model 3 ^d^	0.80 (0.63–1.00)	0.053	1.0	0.64 (0.31–1.33)	0.61 (0.30–1.25)	0.176
Hypo-HDL-cholesterolemia						
Model 1 ^b^	0.89 (0.71–1.11)	0.306	1.0	0.76 (0.46–1.27)	0.76 (0.46–1.27)	0.291
Model 2 ^c^	0.83 (0.66–1.04)	0.103	1.0	0.72 (0.43–1.21)	0.68 (0.40–1.15)	0.149
Model 3 ^d^	0.84 (0.66–1.07)	0.156	1.0	0.77 (0.44–1.34)	0.74 (0.43–1.29)	0.860
Hypertension						
Model 1 ^b^	0.95 (0.78–1.17)	0.627	1.0	1.03 (0.61–1.72)	0.87 (0.53–1.44)	0.582
Model 2 ^c^	0.97 (0.78–1.19)	0.739	1.0	1.05 (0.62–1.76)	0.89 (0.54–1.49)	0.661
Model 3 ^d^	0.96 (0.77–1.20)	0.710	1.0	1.14 (0.66–1.97)	0.89 (0.52–1.51)	0.674
Impaired fasting glucose						
Model 1 ^b^	0.85 (0.71–1.04)	0.097	1.0	0.96 (0.62–1.50)	0.67 (0.43–1.05)	0.079
Model 2 ^c^	0.90 (0.74–1.09)	0.898	1.0	1.01 (0.64–1.58)	0.73 (0.46–1.14)	0.168
Model 3 ^d^	0.92 (0.75–1.13)	0.431	1.0	1.07 (0.66–1.71)	0.76 (0.47–1.22)	0.253

^a^*P* values for trend

^b^Model 1: Crude risk for MetS

^c^Model 2: Adjusted for age and gender

^d^Model 3: Further adjusted for smoking status; alcohol drinking; CRP; and family history of diabetes, hypertension, and CAD

### More significant association between serum Gly levels and MetS in the older groups

**Fig. 3:**
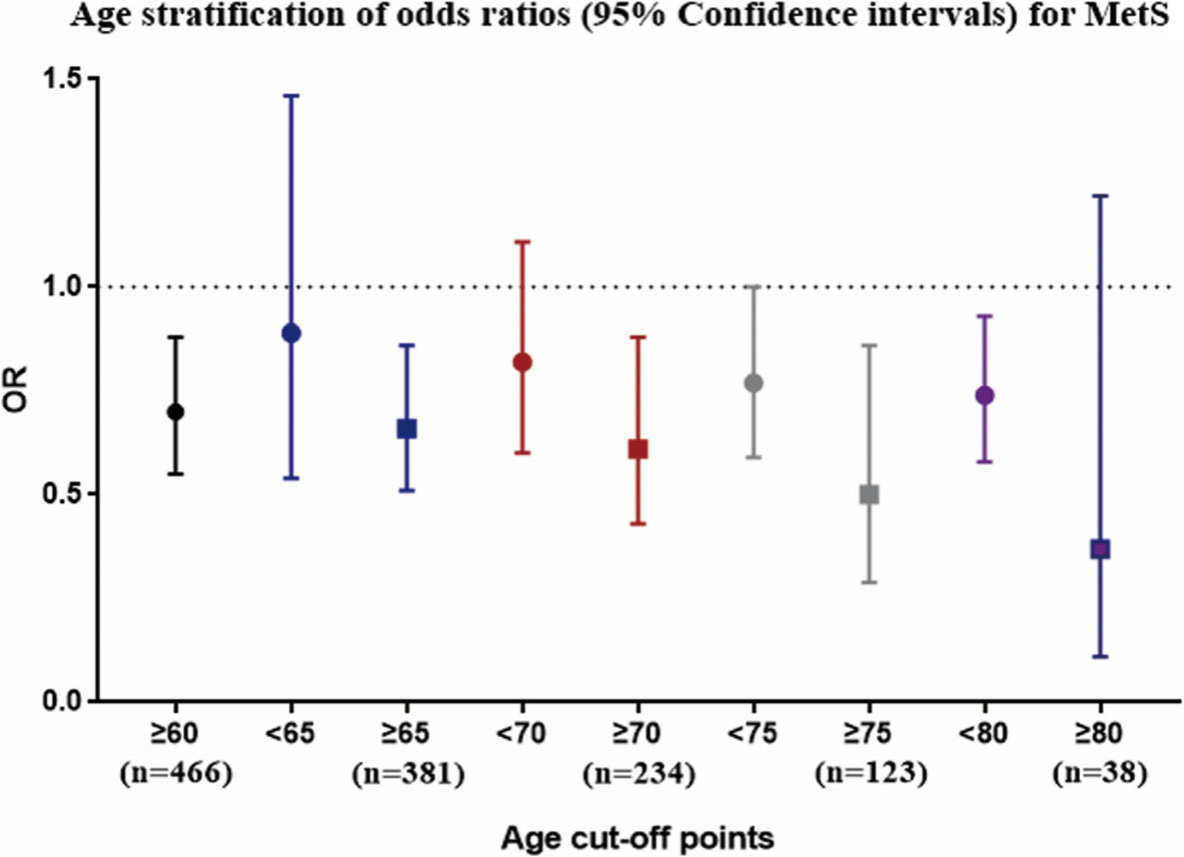
Age stratification of odds ratios (95% confidence intervals) between serum Gly levels and MetS. Adjusted for gender; smoking status; alcohol drinking; CRP; and family history of diabetes, hypertension, and CAD. The y-axis represents the OR of Gly and MetS, and the x-axis represents different age cut-off points

Stratified analysis of the associations of serum Gly levels and MetS was conducted according to a 65-year age cut-off point. The results showed that the OR between serum Gly levels and MetS (95% CI) was 0.71 (0.55–0.91) in the group of those older than 65, whereas in the group of those younger than 65 years old, the OR was 0.92 (0.60–1.42) in model 1, which means that the association was more pronounced in the groups of those aged ≥65 regardless of Gly levels used as a continuous variable or a categorical variable (Tables 4 and 5). What’s more, the ORs for MetS according to serum Gly levels in the groups with ages ≥65 were only a little changed after further adjusting for age and gender (model 2), as well as when additionally controlling for smoking status; alcohol drinking; CRP; and family history of diabetes, hypertension, and CAD (model 3). Further, when we used age stratification analysis at ages 60, 70, 75, and 80, the results also showed that the ORs between serum Gly levels and MetS were more significant in the older groups (Fig. 3).

**Table 4:** Stratified analysis of odds ratios (95% Confidence intervals) for MetS and its components according to serum Gly levels (per 1-SD increment)

	<65y	*P*	≥65y	*P*
	Per SD increment		Per SD increment	
MetS				
Model 1 ^a^	0.92 (0.60–1.42)	0.711	0.71 (0.55–0.91)	0.006
Model 2 ^b^	0.92 (0.60–1.41)	0.695	0.65 (0.50–0.83)	0.001
Model 3 ^c^	0.89 (0.54–1.46)	0.642	0.66 (0.51–0.86)	0.002
Central obesity				
Model 1 ^a^	0.77 (0.50–1.18)	0.236	0.79 (0.64–0.98)	0.028
Model 2 ^b^	0.74 (0.48–1.15)	0.183	0.67 (0.54–0.85)	0.001
Model 3 ^c^	0.69 (0.43–1.12)	0.130	0.67 (0.52–0.86)	0.002
Hypertriglyceridemia				
Model 1 ^a^	1.14 (0.71–1.83)	0.586	0.77 (0.59–0.99)	0.046
Model 2 ^b^	1.11 (0.69–1.78)	0.668	0.70 (0.53–0.91)	0.007
Model 3 ^c^	1.15 (0.70–1.88)	0.574	0.72 (0.55–0.94)	0.016
Hypo-HDL-cholesterolemia				
Model 1 ^a^	1.47 (0.94–2.31)	0.092	0.74 (0.56–0.99)	0.043
Model 2 ^b^	1.44 (0.90–2.28)	0.127	0.69 (0.51–0.92)	0.012
Model 3 ^c^	1.44 (0.88–2.35)	0.145	0.69 (0.51–0.94)	0.018
Hypertension				
Model 1 ^a^	0.97 (0.62–1.53)	0.893	0.95 (0.75–1.19)	0.633
Model 2 ^b^	0.96 (0.60–1.52)	0.850	0.95 (0.75–1.20)	0.679
Model 3 ^c^	0.97 (0.60–1.57)	0.890	0.94 (0.74–1.21)	0.633
Impaired fasting glucose				
Model 1 ^a^	0.98 (0.64–1.51)	0.926	0.83 (0.67–1.02)	0.074
Model 2 ^b^	0.98 (0.63–1.51)	0.924	0.88 (0.70–1.09)	0.232
Model 3 ^c^	0.97 (0.61–1.55)	0.909	0.92 (0.73–1.16)	0.490

^a^Model 1: Crude risk for MetS

^b^Model 2: Adjusted for age and gender

^c^Model 3: Further adjusted for smoking status; alcohol drinking; CRP; and family history of diabetes, hypertension, and CAD

**Table 5:** Stratified analysis of odds ratios (95% Confidence intervals) for MetS and its components according to serum Gly tertiles

	<65y				≥65y			
	T1	T2	T3	*P*_trend_^a^	T1	T2	T3	*P*_trend_^a^
MetS								
Model 1 ^b^	1.0	0.71 (0.25–2.04)	1.08 (0.39–3.04)	0.872	1.0	0.59 (0.36–0.98)	0.34 (0.20–0.58)	< 0.001
Model 2 ^c^	1.0	0.72 (0.25–2.07)	1.09 (0.39–3.05)	0.868	1.0	0.55 (0.3–0.92)	0.29 (0.17–0.51)	< 0.001
Model 3 ^d^	1.0	0.62 (0.18–2.12)	0.96 (0.30–3.01)	0.973	1.0	0.60 (0.35–1.05)	0.31 (0.17–0.56)	< 0.001
Central obesity								
Model 1 ^b^	1.0	0.59 (0.20–1.71)	0.90 (0.30–2.69)	0.862	1.0	0.58 (0.35–0.98)	0.48 (0.29–0.81)	0.006
Model 2 ^c^	1.0	0.62 (0.21–1.82)	0.91 (0.30–2.76)	0.879	1.0	0.52 (0.30–0.89)	0.38 (0.22–0.66)	0.001
Model 3 ^d^	1.0	0.49 (0.15–1.66)	0.73 (0.22–2.43)	0.624	1.0	0.61 (0.35–1.09)	0.41 (0.23–0.73)	0.003
Hypertriglyceridemia								
Model 1 ^b^	1.0	0.51 (0.14–1.77)	0.91 (0.29–2.87)	0.888	1.0	0.67 (0.40–1.14)	0.41 (0.23–0.73)	0.002
Model 2 ^c^	1.0	0.53 (0.15–1.87)	0.92 (0.29–2.29)	0.891	1.0	0.61 (0.35–1.05)	0.34 (0.19–0.62)	< 0.001
Model 3 ^d^	1.0	0.52 (0.14–1.95)	0.98 (0.30–3.18)	0.974	1.0	0.66 (0.37–1.17)	0.38 (0.20–0.70)	0.002
Hypo-HDL-cholesterolemia								
Model 1 ^b^	1.0	1.67 (0.48–5.86)	3.03 (0.90–10.11)	0.066	1.0	0.65 (0.37–1.14)	0.53 (0.29–0.95)	0.032
Model 2 ^c^	1.0	1.87 (0.51–6.82)	3.28 (0.95–11.40)	0.059	1.0	0.60 (0.34–1.07)	0.46 (0.25–0.85)	0.012
Model 3 ^d^	1.0	2.36 (0.54–10.25)	3.98 (0.95–16.60)	0.058	1.0	0.63 (0.34–1.17)	0.51 (0.27–0.97)	0.039
Hypertension								
Model 1 ^b^	1.0	1.10 (0.36–3.32)	1.29 (0.42–3.99)	0.654	1.0	1.01 (0.57–1.81)	0.79 (0.45–1.39)	0.405
Model 2 ^c^	1.0	1.13 (0.37–3.42)	1.30 (0.42–4.01)	0.650	1.0	1.02 (0.57–1.83)	0.79 (0.45–1.41)	0.425
Model 3 ^d^	1.0	1.04 (0.31–3.54)	1.19 (0.35–4.02)	0.784	1.0	1.16 (0.62–2.15)	0.78 (0.43–1.41)	0.393
Impaired fasting glucose								
Model 1 ^b^	1.0	1.61 (0.56–4.64)	1.06 (0.36–3.14)	0.936	1.0	0.87 (0.53–1.42)	0.61 (0.37–1.00)	0.052
Model 2 ^c^	1.0	1.61 (0.56–4.66)	1.06 (0.36–3.15)	0.936	1.0	0.91 (0.56–1.50)	0.67 (0.40–1.10)	0.114
Model 3 ^d^	1.0	1.85 (0.58–5.86)	1.03 (0.33–3.21)	0.992	1.0	0.92 (0.54–1.56)	0.70 (0.41–1.19)	0.182

^a^*P* values for trend

^b^Model 1: Crude risk for MetS

^c^Model 2: Adjusted for age and gender

^d^Model 3: Further adjusted for smoking status; alcohol drinking; CRP; and family history of diabetes, hypertension, and CAD

## Discussion

To the best of our knowledge, this is the first study to estimate the association between serum Gly levels and MetS in the elderly, showing that serum Gly levels declined gradually with increasing prevalence of MetS and number of components of MetS. In addition, after adjusting for age and gender, lower Gly levels were significantly associated with MetS and central obesity. The association was still significant after further controlling for smoking status; alcohol drinking; CRP; and family history of diabetes, hypertension, and CAD. Furthermore, the association between serum Gly levels and MetS was more significant in the older groups.

Several studies have previously reported the negative association between circulating Gly levels and T2D, obesity, and MetS [10, 11, 19]. Similarly, the significant decrease of Gly levels in MetS and obesity is consistent with some prior studies [12, 15]. Serum Gly concentration has been considered the only homeostatic assessment–insulin resistant (HOMA-IR)-associated predictor of both intramuscular adipose tissue and abdominal adiposity in functionally limited overweight older adults [20]. Besides, the associations of Gly levels with other components of MetS, such as hypertension and dyslipidemia, have also been found [13]. However, the current result showing no relationship of Gly levels with these two components of MetS was in contrast to the previous study. The reason for this discrepancy may be due to the minor impact of Gly on these two aspects in the elderly. Moreover, we obtained consistent results when MetS and its components were defined according to other diagnostic standards, such as the Chinese Diabetes Society (2004) and the guidelines for the prevention and treatment of dyslipidemia in Chinese adults (2007).

In the present study, about 80% of subjects were older than 65 years old, and over 98% were older than 60. The chronological age was stratified by the 65-year-old, 70-year-old, 75-year-old, and 80-year-old of age cut-off points. Stratified analysis showed that the association of Gly levels with MetS was more pronounced in the older group than in the relatively younger group. The inflammatory factor, CRP, is also considered a marker of aging, and its lower level may be associated with better survival in the elderly [21, 22]. Recently, inflammatory factors, including CRP, used to evaluate biological age, were proposed by some studies [23, 24]. Therefore, we also conducted a stratified analysis of CRP that represented the biological age and found that the association between Gly levels and MetS was more obvious in the higher hs-CRP group (Table 6), which was consistent with the results of stratified analysis of chronological age. It was demonstrated that it is more important to maintain Gly at high levels in serum in the older group than in the relatively younger group.

**Table 6:** Stratified analysis of odds ratios (95% Confidence intervals) for MetS and its components according to serum Gly tertiles

MetS			
	Model 1 ^b^	Model 2 ^c^	Model 3 ^d^
CRP < 0.09 mg/dl			
T1	1.0	1.0	1.0
T2	0.61 (0.30–1.27)	0.61 (0.30–1.27)	0.64 (0.29–1.41)
T3	0.65 (0.32–1.29)	0.64 (0.32–1.29)	0.64 (0.30–1.34)
*P*_trend_^a^	0.225	0.211	0.799
CRP ≥ 0.09 mg/dl			
T1	1.0	1.0	1.0
T2	0.59 (0.32–1.11)	0.56 (0.29–1.06)	0.66 (0.33–1.31)
T3	0.35 (0.18–0.69)	0.30 (0.15–0.61)	0.32 (0.15–0.68)
*P*_trend_^a^	0.002	0.001	0.003

^a^*P* values for trend

^b^Model 1: Crude risk for MetS

^c^Model 2: Adjusted for age and gender

^d^Model 3: Further adjusted for smoking status; alcohol drinking; CRP; and family history of diabetes, hypertension, and CAD

Our study found that serum Gly concentrations declined gradually with increasing numbers of MetS components and was significantly negatively associated with BMI, WC, TG, and CRP. Therefore, it is beneficial to keep a higher Gly level in elderly people. Several indications demonstrate that Gly supplementation may be a novel therapy for obesity and type 2 diabetes mellitus (T2DM), and dietary supplementation with Gly reduces concentrations of free fatty acids and TG in an animal model of intraabdominal obesity [25]. Moreover, therapy of obesity and T2DM with Gly can improve insulin sensitivity [26], increase anti-inflammatory capacity [27], and normalize secretion of triacylglycerol-rich very-low-density lipoproteins from the liver [28]. Consequently, to hold a higher Gly level in elderly patients with MetS is a new dietary intervention that needs further confirmation.

Based on cross-sectional study of the elderly population, the present study is the first to examine the relationship between Gly levels and MetS and its components in the elderly. However, several limitations should be considered in the future. First, we were unable to obtain causality because of the cross-sectional design of the study. Thus, it is critical to conduct prospective studies in the future. Second, because of the relatively small sample size (the statistical power = 0.86), even though there was a clear trend of the associations of Gly levels and MetS and its components, only MetS and central obesity remained statistically significant after adjustment. Third, we did not record physical activity and other possible risk factors of metabolic diseases in the subjects, so possible confounding of unmeasured factors may exist, even after adjustment.

Our study demonstrated for the first time that serum Gly levels are associated with cardiometabolic characteristics and MetS in the elderly. Patients with MetS have lower Gly levels than do normal subjects. What’s more, the association between Gly levels and MetS is more pronounced in very old people than in younger old people. Our study emphasizes that it is significantly essential for the elderly population to maintain a higher Gly level in serum. Further studies are needed to confirm that Gly is a protective factor for cardiometabolic diseases in the elderly.

## Abbreviations

Apo: Apolipoprotein
CHD: Coronary heart disease
CI: Confidence interval
CRP: High-sensitive C-reactive protein
ESI: Electronic spray ionization
FBG: Fasting blood glucose
Gly: Glycine
HDL-C: High-density lipoprotein cholesterol
HOMA-IR: Homeostatic assessment–insulin resistant
IDF: International Diabetes Federation
IFG: Impaired fasting glucose
LC-MS/MS: Liquid chromatography method associated with mass spectrometry
LDL-C: Low-density lipoprotein cholesterol
MetS: Metabolic syndrome
MRM: Multiple reaction monitoring
OR: Odds ratio
SQRT: Square root
T2D: Type 2 diabetes
TC: Total cholesterol
TG: Triglyceride
WC: Waist circumference
